# Let Continuous Outcome Variables Remain Continuous

**DOI:** 10.1155/2012/639124

**Published:** 2012-05-29

**Authors:** Enayatollah Bakhshi, Brian McArdle, Kazem Mohammad, Behjat Seifi, Akbar Biglarian

**Affiliations:** ^1^Department of Statistics and Computer, University of Social Welfare and Rehabilitation Sciences, Tehran 1985713834, Iran; ^2^Department of Statistics, The University of Auckland, Private Bag 92010, Auckland, New Zealand; ^3^Department of Biostatistics, School of Public Health and Institute of Public Health Research, Tehran University of Medical Sciences, Tehran, Iran; ^4^Department of Physiology, School of Medicine, Tehran University of Medical Sciences, Tehran, Iran

## Abstract

The complementary log-log is an alternative to logistic model. In many areas of research, the outcome data are continuous. We aim to provide a procedure that allows the researcher to estimate the coefficients of the complementary log-log model without dichotomizing and without loss of information. We show that the sample size required for a specific power of the proposed approach is substantially smaller than the dichotomizing method. We find that estimators derived from proposed method are consistently more efficient than dichotomizing method. To illustrate the use of proposed method, we employ the data arising from the NHSI.

## 1. Introduction

Recently, logistic regression has become a popular tool in biomedical studies. The parameter in logistic regression has the interpretation of log odds ratio, which is easy for people such as physicians to understand. Probit and complementary log-log are alternatives to logistic model. For a covariate *X* and a binary response variable *Y*, let *π*(*X*) = *P*(*Y* = 1 | *X* = *x*). A related model to the complementary log-log link is the log-log link. For it, *π*(*x*) approaches 0 sharply but approaches 1 slowly. When the complementary log-log model holds for the probability of a success, the log-log model holds for the probability of a failure [1].

These models use a categorical (dichotomous or polytomous) outcome variable. In many areas of research, the outcome data are continuous. Many researchers have no hesitation in dichotomizing a continuous variable, but this practice does not make use of within-category information. Several investigators have noted the disadvantages of dichotomizing both independent and outcome variables [2–10]. Ragland [11] showed that the magnitude of odds ratio and statistical power depend on the cutpoint used to dichotomize the response variable. From a clinical point of view, binary outcomes may be preferred for some reasons such as (1) setting diagnostic criteria for disease, (2) offering a simpler interpretation of common effect measures from statistical models such as odds ratios and relative risks. However, all advantages come at the lost information. From a statistical point of view, this loss of information means more samples which are required to attain prespecified powers.

Moser and Coombs [12] provided a closed-form relationship that allows a direct comparison between the logistic and linear regression coefficients. They also provided a procedure that allows the researcher to analyze the original continuous outcome without dichotomizing. To date, a method that applies the complementary log-log model without dichotomizing and without loss of information has not been available.

We aim to (a) provide a method that allows the researcher to estimate the coefficients of the complementary log-log model without dichotomizing and without loss of information, (b) show that the coefficient of the complementary log-log model can be interpreted in terms of the regression coefficients, (c) demonstrate that the coefficient estimates from this method have smaller variances and shorter confidence intervals than the dichotomizing method.

## 2. Methods

### 2.1. Model

Let *y*_1_, *y*_2_,…, *y*_*n*_ be *n* independent observations on *y*, and let *x*_1_, *x*_2_,…, *x*_*p*−1_ be *p* − 1 predictor variables thought to be related to the response variable *y*. The multiple linear regression model for the *i*th observation can be expressed as

(1) $$\begin{array}{cc} y_{i} & =\beta_{0}+\beta_{1}x_{i1}+\beta_{2}x_{i2}\\ & \;+\cdots +\beta_{p-1}x_{ip-1}+E_{i}\;i=1,2,\ldots ,n, \end{array}$$

or

(2) $$y_{i}=x_{i}\beta +E_{i}\;i=1,2,\ldots ,n,$$

where

(3) $$x_{i}=\left(1,x_{i1},x_{i2},\ldots ,x_{ip-1}\right).$$

To complete the model, we make the following assumptions:

1. *E*(*E*_*i*_) = 0 for *i* = 1,2,…, *n*,
2. var(*E*_*i*_) = *σ* ^2^ for *i* = 1,2,…, *n*,
3. the independent *E*_*i*_ follows an extreme value distribution for *i* = 1,2,…, *n*.

Writing the model for each of the *n* observations, in matrix form, we have

(4) $$\left[\begin{array}{c} y_{1}\\ y_{2}\\ .\\ .\\ y_{n} \end{array}\right]=\left[\begin{array}{cccc} 1x_{11} & x_{12} & \ldots & x_{1p-1}\\ 1x_{21} & x_{22} & \ldots & x_{2p-1}\\ . & & & \\ . & & & \\ 1x_{n1\;} & x_{n2} & \ldots & x_{np-1} \end{array}\right]\left[\begin{array}{c} \beta_{0}\\ \beta_{1}\\ .\\ .\\ \beta_{p-1} \end{array}\right]+\left[\begin{array}{c} E_{1}\\ E_{2}\\ .\\ .\\ E_{n} \end{array}\right],$$

or

(5) y=Xβ+E.

The preceding three assumptions on *E*_*i*_ and *y*_*i*_  can be expressed in terms of this model:

1. *E*(*E*) = 0,
2. cov⁡(*E*) = *σ* ^2^ *I*,
3. the *E*_*i*_ is extreme value (0, *σ* ^2^) for *i* = 1,2,…, *n*.

### 2.2. (Largest) Extreme Value Distribution

The PDF and CDF of the extreme value distribution are given by

(6) $$\begin{array}{cc} f\left(y\;|\;x\beta ,\sigma \right) & =\frac{\pi }{\sigma \sqrt{6}}\\ & \;\times \exp(-\frac{y-x\beta -k\sigma }{\sigma }\times \frac{\pi }{\sqrt{6}}\\ & \;\;\;\;-\exp\left(\frac{y-x\beta -k\sigma }{\sigma }\times \frac{\pi }{\sqrt{6}}\right))\\ & \;\;-\infty \langle x\langle \infty ,\;\;\sigma \rangle 0,\\ P\left(y\leq c\right) & =\exp\left(-\exp\left(-\frac{c-x\beta +k\sigma }{\sigma }\times \frac{\pi }{\sqrt{6}}\right)\right)\\ & \;-\infty \langle x\langle \infty ,\sigma \rangle ,\;k\approx 0.45. \end{array}$$

It is easy to check that

(7) $$\begin{array}{cc} \omega_{j} & =\frac{\ln\pi_{1}}{\ln\pi_{2}}=\frac{\ln\left(p\left(y\leq c\;|\;x\right)\right)}{\ln\left(p\left(y\leq c\;|\;x_{\left(-1,j\right)}\right)\right)}\\ & =\frac{-\exp\left(-\left(\left(c-x^{\prime }\beta +k\sigma \right)/\sigma \right)\times \pi /\sqrt{6}\right)}{-\exp\left(-\left(\left(c-x_{\left(-1,j\right)}^{\prime }\beta +k\sigma \right)/\sigma \right)\times \pi /\sqrt{6}\right)}\\ & =\exp\left(\frac{\pi }{\sqrt{6}}\cdot \frac{\beta_{j}}{\sigma }\right)\Rightarrow \pi_{1}={\pi_{2}}^{\exp(\left(\pi /\sqrt{6})\cdot (\beta_{j}/\sigma \right))}, \end{array}$$

where

(8) $$\begin{array}{cc} x & =\left(1,x_{1},\ldots ,x_{j},\ldots ,x_{p-1}\right),\\ x_{\left(-1,j\right)} & =\left(1,x_{1},\ldots ,x_{j}-1,\ldots ,x_{p-1}\right),\\ \beta & ={\left(\beta_{0},\;\beta_{1},\ldots ,\beta_{j},\ldots ,\beta_{p-1}\right)}^{\prime }. \end{array}$$

To return to a random sample of observations (*y*_1_, *y*_2_,…, *y*_*n*_), we conclude that the PDF and CDF of each independent *y*_*i*_ are given by (6), and the corresponding equality (7) is given by

(9) $$\frac{\ln{\hat{\pi }}_{1}}{\ln{\hat{\pi }}_{2}}=\exp\left(\frac{\pi }{\hat{\sigma }\sqrt{6}}{\hat{\beta }}_{j}\right),$$

where the estimate ${\hat{\beta }}_{j}\;$is the (*j* + 1)th element of vector $\hat{\beta }={\left({\hat{\beta }}_{0},{\hat{\beta }}_{1},\ldots ,{\hat{\beta }}_{j},\ldots ,{\hat{\beta }}_{p-1}\right)}^{\prime }$. It is readily shown that the results also hold true for the smallest extreme value distribution (Appendix A).

### 2.3. The Proposed Confidence Intervals

 Let

(10) $$\begin{array}{cc} \hat{\beta } & ={\left({\hat{\beta }}_{0},{\hat{\beta }}_{1},\ldots ,{\hat{\beta }}_{j},\ldots ,{\hat{\beta }}_{p-1}\right)}^{\prime }\\ & ={\left(X^{\prime }X\right)}^{-1}X^{\prime }Y\;j=0,\ldots ,p-1,\\ {\hat{\sigma }}^{2} & =\frac{Y^{\prime }\left(I_{n}-X{\left(X^{\prime }X\right)}^{-1}X^{\prime }\right)Y}{\left(n-p\right)}. \end{array}$$

According to the preceding three assumptions on *E*_*i*_ and  *y*_*i*_, we obtain

(11) $$\begin{array}{cc} E\left(\hat{\beta }\right) & =E\left[{\left(X^{\prime }X\right)}^{-1}X^{\prime }Y\right]\\ & ={\left(X^{\prime }X\right)}^{-1}X^{\prime }EY={\left(X^{\prime }X\right)}^{-1}X^{\prime }X\beta =\beta ,\\ E\left({\hat{\sigma }}^{2}\right) & =\frac{1}{n-p}E\left(Y^{\prime }\left(I_{n}-X{\left(X^{\prime }X\right)}^{-1}X^{\prime }\right)Y\right)\\ & =\frac{1}{n-p}\{\mathrm{tr}\left[\left(I_{n}-X{\left(X^{\prime }X\right)}^{-1}X^{\prime }\right)\sigma^{2}I\right]\\ & \;\;\;\;+E\left(Y^{\prime }\right)\left[I_{n}-X{\left(X^{\prime }X\right)}^{-1}X^{\prime }\right]E\left(Y\right)\}\\ & =\frac{1}{n-p}\{\sigma^{2}\mathrm{tr}\left[I_{n}-X{\left(X^{\prime }X\right)}^{-1}X^{\prime }\right]\;\;\\ & \;\;\;\;+\beta^{\prime }X^{\prime }\left[I_{n}-X{\left(X^{\prime }X\right)}^{-1}X^{\prime }\right]X\beta \}\\ & =\frac{1}{n-p}\{\sigma^{2}\left[n-\mathrm{tr}\left(X{\left(X^{\prime }X\right)}^{-1}X^{\prime }\right)\right]\\ & \;\;\;\;+\beta^{\prime }X^{\prime }X\beta -\beta^{\prime }X^{\prime }X{\left(X^{\prime }X\right)}^{-1}X^{\prime }X\beta \}\\ & =\frac{1}{n-p}\{\sigma^{2}\left[n-\mathrm{tr}\left(X{\left(X^{\prime }X\right)}^{-1}X^{\prime }\right)\right]\\ & \;\;\;\;+\beta^{\prime }X^{\prime }X\beta -\beta^{\prime }X^{\prime }X\beta \}\\ & =\frac{1}{n-p}\sigma^{2}\left[n-\mathrm{tr}\left(I_{P}\right)\right]=\frac{1}{n-p}\sigma^{2}\left(n-p\right)=\sigma^{2}. \end{array}$$

Therefore, $\hat{\beta }$ and$\;{\hat{\sigma }}^{2}\;$are unbiased estimators of *β* and  *σ* ^2^.

We have assumed that *E*_*i*_ is distributed as an extreme value, and we use the approximation of the extreme value distribution of the errors *E*_*i*_ by the normal distribution. For normally distributed observations, ${\hat{\beta }}_{j}/\left(\hat{\sigma }\sqrt{\delta_{j}}\right)$ follows a noncentral *t* distribution with *n* − *p* degree of freedom and noncentrality parameter $-\infty <\beta_{j}/\left(\sigma \sqrt{\delta_{j}}\right)<\infty$,

(12) $$\begin{array}{cc} 1-\alpha & =P\{t_{1-(\alpha /2)}\left[n-p,\frac{\beta_{j}}{\left(\sigma \sqrt{\delta_{j}}\right)}\right]\\ & \;\;<\frac{{\hat{\beta }}_{j}}{\left(\hat{\sigma }\sqrt{\delta_{j}}\right)}<t_{\alpha /2}\left[n-p,\frac{\beta_{j}}{\left(\sigma \sqrt{\delta_{j}}\right)}\right]\}, \end{array}$$

where *t*_*α*/2_[*r*, *s*] represents the 100(1 − (*α*/2))  percentile point of a noncentral *t* distribution with *r* degrees of freedom and noncentrality parameter −*∞* < *s* < *∞*, and *δ*_*j*_ is the (*j* + 1)st diagonal element of (*X*′*X*)^−1^. We use the approximation of the percentiles of the noncentral *t* distribution by the standard normal percentiles [13], then

(13) $$\begin{array}{cc} 1-\alpha & =P\left\{\begin{array}{cc} & \frac{\beta_{j}/\left(\sigma \sqrt{\delta_{j}}\right)-z_{\alpha /2}{\left[1+\left(\beta_{j}^{2}/\left(\sigma^{2}\delta_{j}\right)-z_{\alpha /2}^{2}\right)/2\left(n-p\right)\right]}^{1/2}}{1-\left(z_{\alpha /2}^{2}/2\left(n-p\right)\right)\;}<\\ & \frac{{\hat{\beta }}_{j}}{\left(\hat{\sigma }\sqrt{\delta_{j}}\right)}<\frac{\beta_{j}/\left(\sigma \sqrt{\delta_{j}}\right)+z_{\alpha /2}{\left[1+\left(\left(\beta_{j}^{2}/\left(\sigma^{2}\delta_{j}\right)-z_{\alpha /2}^{2}\right)/2\left(n-p\right)\right)\right]}^{1/2}}{1-\left(z_{\alpha /2}^{2}/2\left(n-p\right)\right)\;} \end{array}\right\},\\ {\left(\frac{\beta_{j}}{\sigma }\right)}^{U} & =\left\{\frac{{\hat{\beta }}_{j}}{\hat{\sigma }}\left[1-\frac{z_{\alpha /2}^{2}}{2\left(n-p\right)}\right]+z_{\alpha /2}{\left[\delta_{j}\left(1+\left(\frac{\left({\hat{\beta }}_{j}^{2}/{\hat{\sigma }}^{2}\delta_{j}\right)-z_{\alpha /2}^{2}}{2\left(n-p\right)}\right)\right)\right]}^{1/2}\right\},\\ {\left(\frac{\beta_{j}}{\sigma }\right)}^{L} & =\left\{\frac{{\hat{\beta }}_{j}}{\hat{\sigma }}\left[1-\frac{z_{\alpha /2}^{2}}{2\left(n-p\right)}\right]-z_{\alpha /2}{\left[\delta_{j}\left(1+\left(\frac{\left({\hat{\beta }}_{j}^{2}/{\hat{\sigma }}^{2}\delta_{j}\right)-z_{\alpha /2}^{2}}{2\left(n-p\right)}\right)\right)\right]}^{1/2}\right\}, \end{array}$$

Thus, we obtain an approximate 100(1 − *α*) percent confidence interval for *ω*_*j*_

(14) $$\left\{\exp\left[\frac{\pi }{\sqrt{6}}{\left(\frac{\beta_{j}}{\sigma }\right)}^{L}\right],\;\exp{\left[\frac{\pi }{\sqrt{6}}{\left(\frac{\beta_{j}}{\sigma }\right)}^{U}\right]}^{}\right\}.$$

## 3. Comparison of the Two Methods

Let *Y*_*i*_ be a continuous outcome variable. For fixed value of *C*, we define *Y*_i_* such that

(15) $$Y_{i}^{*}=\{\begin{array}{c} 1\;\text{if}\;Y_{i}^{}\geq C,\\ 0\;\text{if}\;{Y_{i}}^{}<C. \end{array}$$

Suppose that *Y*_1_*,…, *Y*_n_* form a random sample of observations, and we fit a complementary log-log model

(16) $$\begin{array}{c} \pi_{i1}=P\left(Y_{i}^{*}=1\;|\;x_{i}\right)=\exp\left(-\exp\left(x_{i}^{}\theta \right)\right),\\ \pi_{i2}=P\left(Y_{i}^{*}=1\;|\;x_{\left(-1,i\right)}\right)=\exp\left(-\exp\left(x_{\left(-1,i\right)}\theta \right)\right), \end{array}$$

where *x*_*i*_ = (1,*x*_*i*1_,…,*x*_*i*,*p*−1_)′  is the *P* × 1 vector of covariates for the *i*th observation, and *θ* = (*θ*_0_,…,*θ*_*p*−1_)′ is the *P* × 1 vector of unknown parameters. The dichotomized *ω*_j_* parameter corresponding to the effect *θ*_*j*_ is

(17) $$\begin{array}{cc} \omega_{j}^{*} & =\frac{\ln\left(\pi_{1}\right)}{\ln\left(\pi_{2}\right)}\\ & =\frac{\ln\left(P\left(Y^{*}=1\;|\;x\right)\right)}{\ln\left(P\left(Y^{*}=1\;|\;x_{\left(-1,j\right)}\right)\right)}\\ & =\frac{\left(\exp\left(x\theta \right)\right)}{\left(\exp\left(x_{\left(-1,j\right)}\theta \right)\right)}\\ & =\exp\left(\theta_{j}\right)\;j=0,\ldots ,p-1. \end{array}$$

In general, maximum likelihood estimation (MLE) can be used to estimate the parameter  *θ* = (*θ*_0_,…, *θ*_*p*−1_). Let $\hat{\theta }={\left({\hat{\theta }}_{0},\ldots ,{\hat{\theta }}_{p-1}\right)}^{\prime }$ be the *P* × 1 ML estimate of *θ*, and let $\text{COV}\left(\hat{\theta }\right)$ be the *P* × *P*  covariance matrix of $\hat{\theta }$. Using $\text{COV}\left(\hat{\theta }\right)$ from (23), one can construct confidence intervals. This matrix has as its diagonal the estimated variances of each of the ML estimates. The (*j* + 1)th diagonal element is given by $\sigma_{{\hat{\theta }}_{j}}^{2}$. Therefore,

(18) $${\hat{\omega }}_{j}^{*}=\exp\left({\hat{\theta }}_{j}\right),$$

and for large samples, $\left({\hat{\theta }}_{j}^{L},{\hat{\theta }}_{j}^{U}\right)=\left({\hat{\theta }}_{j}-z_{\alpha /2}{\hat{\sigma }}_{{\hat{\theta }}_{j}},{\hat{\theta }}_{j}+z_{\alpha /2}{\hat{\sigma }}_{{\hat{\theta }}_{j}}\right)$ is a 100(1 − *α*) percent confidence interval for the true *θ*_*j*_. Then$\;\left(\exp\left({\hat{\theta }}_{j}^{L}\right),\exp\left({\hat{\theta }}_{j}^{U}\right)\right)$ is a 100(1 − *α*) percent confidence interval for the true *ω*_j_*.

We now compare the *ω*_*j*_ from (7) with the *ω*_j_* from (17)

(19) $$\begin{array}{cc} \begin{array}{c} \omega_{j}=\frac{\ln\left(\pi_{1}\right)}{\ln\left(\pi_{2}\right)}\\ \omega_{j}^{*}=\frac{\ln\left(\pi_{1}\right)}{\ln\left(\pi_{2}\right)} \end{array} & \Rightarrow \omega_{j}^{*}=\omega_{j}\\ & \Rightarrow \exp\left(\frac{\pi }{\sqrt{6}}\cdot \frac{\beta_{j}}{\sigma }\right)=\exp\left(\theta_{j}\right)\\ & \Rightarrow \frac{\pi }{\sqrt{6}}\cdot \frac{\beta_{j}}{\sigma }=\theta_{j}\;\forall \beta_{j},\theta_{j},\sigma . \end{array}$$

This show that the coefficient of the complementary log-log model, *θ*_*j*_, can be interpreted in terms of the regression coefficients, *β*_*j*_. Note that *β* are related to the responses through the general linear regression model

(20) $$y_{i}=x_{i}\beta +E_{i}\;i=1,\ldots ,n,$$

where the independent *E*_*i*_ are distributed as an extreme value with mean 0 and variance *σ* ^2^ > 0.

## 4. Covariance Matrix of Model Parameter Estimators

### 4.1. Derivation of var(*ω*_j_*) for Large *n*

 The information matrix of generalized linear models has the form ∫ = *X*′*WX* [1], where *W* is the diagonal matrix with diagonal elements *w*_*i*_ = (∂*μ*_*i*_/∂*η*_*i*_)^2^/(var(*y*_*i*_)), *y* is response variable with independent observations (*y*_1_,…*y*_*n*_), and *x*_*ij*_  denote the value of predictor *j*,

(21) $$\mu_{i}=E\left(y_{i}\right),\;\;\eta_{i}=g\left(\mu_{i}\right)=\sum_{j}\theta_{j}x_{ij},\;j=0,1,\ldots ,p-1.$$

The covariance matrix of $\hat{\theta }$ is estimated by$\;{\left(X^{\prime }\hat{W}X\right)}^{-1}$.

Maximum likelihood estimation for the complementary log-log model is a special case of the generalized linear models. Let

(22) $$\begin{array}{cc} \mu_{i} & =\pi_{i}=\exp\left(-\exp\left(\sum_{j}\theta_{j}x_{ij}\right)\right)\\ & \Rightarrow \pi_{i}=\exp\left(-\exp\left(\eta_{i}\right)\right),\\ \frac{\partial \mu_{i}}{\partial \eta_{i}} & ={\left(-\exp\left(\eta_{i}\right)\right)}^{\prime }\exp\left(-\exp\left(\eta_{i}\right)\right)=\pi_{i}\ln\pi_{i},\\ w_{i} & =\frac{{\left(\pi_{I}\ln\pi_{i}\right)}^{2}}{\pi_{i}\left(1-\pi_{i}\right)}=\frac{\pi_{i}{\left(\ln\pi_{i}\right)}^{2}}{1-\pi_{i}}, \end{array}$$

then

(23) $$X^{\prime }WX=\left[\begin{array}{cccc} \overset{n}{\underset{i=1}{\sum }}\frac{\pi_{i}{\left(\ln\pi_{i}\right)}^{2}}{1-\pi_{i}} & \overset{n}{\underset{i=1}{\sum }}x_{i1}\frac{\pi_{i}{\left(\ln\pi_{i}\right)}^{2}}{1-\pi_{i}} & \cdots & \overset{n}{\underset{i=1}{\sum }}x_{i,p-1}\frac{\pi_{i}{\left(\ln\pi_{i}\right)}^{2}}{1-\pi_{i}}\\ \overset{n}{\underset{i=1}{\sum }}x_{i1}\frac{\pi_{i}{\left(\ln\pi_{i}\right)}^{2}}{1-\pi_{i}} & \overset{n}{\underset{i=1}{\sum }}x_{i1}^{2}\frac{\pi_{i}{\left(\ln\pi_{i}\right)}^{2}}{1-\pi_{i}} & \cdots & \overset{n}{\underset{i=1}{\sum }}x_{1}x_{i,p-1}\frac{\pi_{i}{\left(\ln\pi_{i}\right)}^{2}}{1-\pi_{i}}\\ \vdots & & \vdots & \\ \overset{n}{\underset{i=1}{\sum }}x_{i,p-1}\frac{\pi_{i}{\left(\ln\pi_{i}\right)}^{2}}{1-\pi_{i}} & \overset{n}{\underset{i=1}{\sum }}x_{1i}x_{i,p-1}\frac{\pi_{i}{\left(\ln\pi_{i}\right)}^{2}}{1-\pi_{i}} & \cdots & \overset{n}{\underset{i=1}{\sum }}x_{i,p-1}^{2}\frac{\pi_{i}{\left(\ln\pi_{i}\right)}^{2}}{1-\pi_{i}} \end{array}\right].$$

It is readily shown that the results hold true for the largest extreme value distribution (Appendix A).

In large samples, $\text{var}\left({\hat{\theta }}_{j}\right)$ approaches $\sigma_{\theta_{j}}^{2}|{}_{\theta =\hat{\theta }}$ [14] which equals the (*j* + 1)th diagonal element of (*X*′*WX*)^−1^.

By applying the delta method, let $f\left({\hat{\theta }}_{j}\right)=\exp\left({\hat{\theta }}_{j}\right)$, then

(24) $$\begin{array}{cc} \text{var}\left({\hat{\omega }}_{j}^{*}\right)\to \text{var}\left(\exp\left({\hat{\theta }}_{j}\right)\right) & =\text{var}\left(f\left({\hat{\theta }}_{j}\right)\right)\\ & ={\left(\frac{\partial f\left({\hat{\theta }}_{j}\right)}{\partial {\hat{\theta }}_{j}}|{}_{{\hat{\theta }}_{j}=\theta_{j}}\right)}^{2}\left(\text{var}\left({\hat{\theta }}_{j}\right)\right)\\ & ={\left(\exp\left(\theta_{j}\right)\right)}^{2}\times \sigma_{{\hat{\theta }}_{j}}^{2}. \end{array}$$

### 4.2. Derivation of $\text{var}\left({\hat{\omega }}_{j}\right)$ for Large *n*

In large samples, from (10) ${\hat{\sigma }}^{2}\to \sigma^{2}$ [15]. Therefore,

(25) $$\text{var}\left({\hat{\omega }}_{j}\right)=\text{var}\left(\exp\left(\frac{\pi {\hat{\beta }}_{j}}{\hat{\sigma }\sqrt{6}}\right)\right)\to \text{var}\left(\exp\left(\frac{\pi {\hat{\beta }}_{j}}{\sigma \sqrt{6}}\right)\right).$$

In addition, $\text{var}\left({\hat{\beta }}_{j}\right)=\sigma^{2}\delta_{j}$.

By applying the delta method, let $g\left({\hat{\beta }}_{j}\right)=\exp\left(\pi {\hat{\beta }}_{j}/\left(\sigma \sqrt{6}\right)\right)$, then

(26) $$\begin{array}{cc} & \text{var}\left({\hat{\omega }}_{j}\right)\to \text{var}\left(\exp\left(\frac{\pi {\hat{\beta }}_{j}}{\sigma \sqrt{6}}\right)\right)\\ & \;\;=\text{var}\left(g\left({\hat{\beta }}_{j}\right)\right)\\ & \;\;={\left(\frac{\partial g\left({\hat{\beta }}_{j}\right)}{\partial {\hat{\beta }}_{j}}|{}_{{\hat{\beta }}_{j}=\beta_{j}}\right)}^{2}\times \text{var}\left({\hat{\beta }}_{j}\right)\\ & \;\;={\left(\frac{\pi }{\sigma \sqrt{6}}\exp\left(\frac{\pi \beta_{j}}{\sigma \sqrt{6}}\right)\right)}^{2}\sigma^{2}\delta_{j}\\ & \;\;=\frac{\pi^{2}}{\sqrt{6}}\delta_{j}{\left(\exp\frac{\pi \beta_{j}}{\sigma \sqrt{6}}\right)}^{2}. \end{array}$$

## 5. Sample Sizes Saving

### 5.1. The Power for the Dichotomized Method

In large samples, ${\hat{\sigma }}_{{\hat{\theta }}_{j}}$ converges to $\sigma_{{\hat{\theta }}_{j}}$ almost surely [14]. Therefore, for a given value of *ω*_*j*_ = exp⁡*θ*_*j*_ (i.e., ln⁡*ω*_*j*_ = *θ*_*j*_), the power is given by

(27) $$\begin{array}{cc} p\left(\omega_{j}\right) & =p\left\{\text{rejection}\;\text{of}\;\omega_{j}=1\;|\;\omega_{j}\neq 1\right\}\\ & =p\left\{\exp\left(\theta_{j}^{L}\right)>1\;|\;\theta_{j}\right\}+p\left\{\exp\left(\theta_{j}^{U}\right)<1\;|\;\theta_{j}\right\}\\ & =p\left\{{\hat{\theta }}_{j}>z_{\alpha /2}\sigma_{{\hat{\theta }}_{j}}\;|\;\theta_{j}\right\}+p\left\{{\hat{\theta }}_{j}<-z_{\alpha /2}\sigma_{{\hat{\theta }}_{j}}\;|\;\theta_{j}\right\}\\ & =p\left\{Z>\frac{z_{\alpha /2}\sigma_{{\hat{\theta }}_{j}}-\ln\omega_{j}}{\sigma_{{\hat{\theta }}_{j}}}\right\}\\ & \;+p\left\{Z<\frac{-z_{\alpha /2}\sigma_{{\hat{\theta }}_{j}}-\ln\omega_{j}}{\sigma_{{\hat{\theta }}_{j}}}\right\}\\ & =p\left\{Z>z_{\alpha /2}-\frac{\ln\omega_{j}}{\sigma_{{\hat{\theta }}_{j}}}\right\}+p\left\{Z<-z_{\alpha /2}-\frac{\ln\omega_{j}}{\sigma_{{\hat{\theta }}_{j}}}\right\}\\ & =P\left\{Z>z_{1}^{•}\right\}+P\left\{Z<-z_{2}^{•}\right\}, \end{array}$$

where

(28) $$\left\{\begin{array}{c} z_{1}^{•}=z_{\alpha /2}-\frac{\ln\omega_{j}}{\sigma_{{\hat{\theta }}_{j}}}\\ z_{2}^{•}=z_{\alpha /2}+\frac{\ln\omega_{j}}{\sigma_{{\hat{\theta }}_{j}}} \end{array}\right\}.$$

### 5.2. The Power for the Proposed Method

In large samples, $\hat{\sigma }$ converges to *σ* almost surely [15]. Therefore, for a given value of $\omega_{j}=\exp\left(\pi \beta_{j}/\sigma \sqrt{6}\right)$ (i.e., $\beta_{j}=\sigma (\ln\omega_{j}\sqrt{6}/\pi )$), the power is given by

(29) $$\begin{array}{cc} p\left(\omega_{j}\right) & =p\left\{\exp\left(\frac{\pi }{\sqrt{6}}{\left(\frac{\beta_{j}}{\sigma }\right)}^{L}\right)>1\;|\;\omega_{j}\right\}\\ & \;+p\left\{\exp\left(\frac{\pi }{\sqrt{6}}{\left(\frac{\beta_{j}}{\sigma }\right)}^{U}\right)<1\;|\;\omega_{j}\right\}\\ & =P\left\{\beta_{J}^{L}>z_{\alpha /2}\sigma \sqrt{\delta_{j}}\;|\;\beta_{j}=\frac{\sigma \ln\omega_{j}\sqrt{6}}{\pi }\right\}\\ & \;+P\left\{\beta_{J}^{U}<-z_{\alpha /2}\sigma \sqrt{\delta_{j}}\;|\;\beta_{j}=\frac{\sigma \ln\omega_{j}\sqrt{6}}{\pi }\right\}\\ & =p\left\{Z>\frac{z_{\alpha /2}\sigma \sqrt{\delta_{j}}-\left(\sigma \ln\omega_{j}\sqrt{6}/\pi \right)}{\sigma \sqrt{\delta_{j}}}\right\}\\ & \;+p\left\{Z<\frac{-z_{\alpha /2}\sigma \sqrt{\delta_{j}}-\left(\sigma \ln\omega_{j}\sqrt{6}/\pi \right)}{\sigma \sqrt{\delta_{j}}}\right\}\\ & =p\left\{Z>z_{\alpha /2}-\frac{\ln\omega_{j}\sqrt{6}}{\pi \sqrt{\delta_{j}}}\right\}\\ & \;+p\left\{Z<-z_{\alpha /2}-\frac{\ln\omega_{j}\sqrt{6}}{\pi \sqrt{\delta_{j}}}\right\}\\ & =p\left\{Z>z_{1}\right\}+p\left\{Z<-z_{2}\right\}, \end{array}$$

where

(30) $$\;\left\{\begin{array}{c} z_{1}=z_{\alpha /2}-\frac{\ln\omega_{j}\sqrt{6}}{\pi \sqrt{\delta_{j}}}\\ z_{2}=z_{\alpha /2}+\frac{\ln\omega_{j}\sqrt{6}}{\pi \sqrt{\delta_{j}}} \end{array}\right\}.$$

Our proposed method, since it is based on continuous data rather than dichotomized, is likely to be more powerful. We show that the proposed method can produce substantial sample size saving for a given power. Let

- the number of parameters *p* = 2 (i.e., *θ* = (*θ*_0_, *θ*_1_)),
- *x*_*i*_ = (1,*x*_*i*1_)′, *x*_*i*1_ ∈ {−*a* + (2*an*/(*g* − 1)) | *n* = 0,…, *g* − 1}, that is, *x*_*i*1_  follows a discrete uniform distribution with range (−*a*, *a*). For simplicity, *a* = 2.
- Total samples are *n* and n* for the proposed and dichotomized methods, respectively. These samples included *k* and k* set of these *g* uniformly distributed points for the proposed and dichotomized methods, respectively. That is, *n* = *gk* and n* = gk*, then

(31) $$\delta_{j}={\left[k\overset{g}{\underset{i=1}{\sum }}{\left(x_{1i}-{\overset{̅}{x}}_{1.}\right)}^{2}\right]}^{-1},\;j=1,$$

and from (23),

(32) $$\sigma_{{\hat{\theta }}_{j}}^{2}=\frac{\sum_{i=1}^{g}\left(\left(\pi_{i}\right){\left(\ln\left(\pi_{i}\right)\right)}^{2}/\ln\left(1-\pi_{i}\right)\right)}{\left(k^{*}\right)\left\{\sum_{i=1}^{g}x_{1i}^{2}\left(\left(\pi_{i}\right){\left(\ln\left(\pi_{i}\right)\right)}^{2}/\ln\left(1-\pi_{i}\right)\right)\sum_{i=1}^{g}\left(\left(\pi_{i}\right){\left(\ln\left(\pi_{i}\right)\right)}^{2}/\ln\left(1-\pi_{i}\right)\right)-{\left[\sum_{i=1}^{g}x_{1i}\left(\left(\pi_{i}\right){\left(\ln\left(\pi_{i}\right)\right)}^{2}/\ln\left(1-\pi_{i}\right)\right)\right]}^{2}\right\}}.$$

We consider the same power for two methods:

(33) $$\begin{array}{cc} & \begin{array}{c} z_{1}=z_{1}^{*}\\ z_{2}=z_{2}^{*} \end{array}\Rightarrow \{\begin{array}{c} z_{\alpha /2}-\frac{\ln\omega_{j}}{\sigma_{{\hat{\theta }}_{j}}}=z_{\alpha /2}-\frac{\ln\omega_{j}\sqrt{6}}{\pi \sqrt{\delta_{j}}}\\ z_{\alpha /2}+\frac{\ln\omega_{j}}{\sigma_{{\hat{\theta }}_{j}}}=z_{\alpha /2}+\frac{\ln\omega_{j}\sqrt{6}}{\pi \sqrt{\delta_{j}}} \end{array}\Rightarrow \frac{\pi }{\sqrt{6}}\sqrt{\delta_{j}}=\sigma_{{\hat{\theta }}_{j}},\;j=1\Rightarrow \frac{\pi }{\sqrt{6}}\sqrt{{\left[k\overset{g}{\underset{i=1}{\sum }}{\left(x_{1i}-{\overset{̅}{x}}_{1.}\right)}^{2}\right]}^{-1}}\\ & =\sqrt{\frac{\sum_{i=1}^{g}\left(\left(\pi_{i}\right){\left(\ln\left(\pi_{i}\right)\right)}^{2}/\ln\left(1-\pi_{i}\right)\right)}{\left(k^{*}\right)\left\{\sum_{i=1}^{g}x_{1i}^{2}\left(\left(\pi_{i}\right){\left(\ln\left(\pi_{i}\right)\right)}^{2}/\ln\left(1-\pi_{i}\right)\right)\sum_{i=1}^{g}\left(\left(\pi_{i}\right){\left(\ln\left(\pi_{i}\right)\right)}^{2}/\ln\left(1-\pi_{i}\right)\right)-{\left[\sum_{i=1}^{g}x_{1i}\left(\left(\pi_{i}\right){\left(\ln\left(\pi_{i}\right)\right)}^{2}/\ln\left(1-\pi_{i}\right)\right)\right]}^{2}\right\}}} \end{array}$$

relative sample size

(34) $$\begin{array}{cc} \frac{n^{*}}{n} & =\frac{k^{*}}{k}=\frac{6\sigma_{{\hat{\theta }}_{j}}^{2}}{\pi^{2}\delta_{j}}\\ & =\frac{\sum_{i=1}^{g}{\left(x_{1i}-{\overset{̅}{x}}_{1.}\right)}^{2}\times \sum_{i=1}^{g}\left(\left(\pi_{i}\right){\left(\ln\left(\pi_{i}\right)\right)}^{2}/\ln\left(1-\pi_{i}\right)\right)}{\left(\pi^{2}/6\right)\left\{\sum_{i=1}^{g}x_{1i}^{2}\left(\left(\pi_{i}\right){\left(\ln\left(\pi_{i}\right)\right)}^{2}/\ln\left(1-\pi_{i}\right)\right)\sum_{i=1}^{g}\left(\left(\pi_{i}\right){\left(\ln\left(\pi_{i}\right)\right)}^{2}/\ln\left(1-\pi_{i}\right)\right)-{\left[\sum_{i=1}^{g}x_{1i}\left(\left(\pi_{i}\right){\left(\ln\left(\pi_{i}\right)\right)}^{2}/\ln\left(1-\pi_{i}\right)\right)\right]}^{2}\right\}}. \end{array}$$

That is, (34) is independent of *σ* ^2^ and applies for any power, and any test size *α*.

Table 1 presents relative sample sizes n*/*n* for a given fixed parameter *ω*_j_*  and an average proportion of success $\overset{̅}{\pi }$. We consider the situations in which $\overset{̅}{\pi }=\sum_{i=1}^{g}\left(\pi_{i}/g\right)=0.1,0.2,0.3,0.4,0.5$, *g* = 9, *ω*_j_* = 0.25,0.50,0.75. 

For given fixed *ω*_j_* and $\overset{̅}{\pi }$, the relative sample sizes in Table 1 can be computed by the following step:

- compute the value *θ*_*j*_ via the equation *θ*_*j*_ = ln⁡(*ω*_j_*),
- calculate the cut-off point *C* iteratively such that $\overset{̅}{\pi }$ attained the specified value for the values *x*_*i*1_, using the value of *θ*_*j*_  in (i).

As can be seen from Table 1, all values are greater than 1. The values of n*/*n* increase as the  *ω*_j_* moves farther away from 1. Values of Table 1 immediately highlight the improvement accomplished by the proposed method.

## 6. Relative Efficiency of ${\hat{\omega }}_{j}$ with ${\hat{\omega }}_{j}^{*}$

Here, we examine the relative efficiency of the estimate$\;{\hat{\omega }}_{j}$ to the estimate ${\hat{\omega }}_{j}^{*}$.

Using (24) and (26), the relative efficiency is given by

(35) $$\begin{array}{cc} \text{r}.\text{e}.\;\left({\hat{\omega }}_{j},{\hat{\omega }}_{j}^{*}\right) & =\frac{\text{var}\left({\hat{\omega }}_{j}^{*}\right)}{\text{var}\left({\hat{\omega }}_{j}\right)}\\ & =\frac{6{\left(\exp\left(\theta_{j}\right)\right)}^{2}\times \sigma_{{\hat{\theta }}_{j}}^{2}}{\pi^{2}\delta_{j}{\left(\exp\left(\lambda \beta_{j}/\sigma \right)\right)}^{2}}=\frac{6\sigma_{{\hat{\theta }}_{j}}^{2}}{\pi^{2}\delta_{j}}. \end{array}$$

Note that the relative efficiency is independent of *n* and *σ* ^2^ and converges to a constant. Comparing (34) and (35), the relative efficiency equals the relative sample sizes. Therefore, as in Table 1, the proposed method is a consistent improvement over the dichotomizing method with respect to relative efficiencies.

It should be noted that these results hold true under the following assumptions:

1. the responses *y*_*i*_ and *β* are related through the equation *y*_*i*_ = *x*_*i*_ *β* + *E*_*i*_ where the independent  *E*_*i*_ are distributed as an extreme value with mean 0 and variance *σ* ^2^ > 0,
2. the independent variables *x*_*i*_ follow a discrete uniform distribution.

## 7. Odds Ratio

For values of *π* larger than 0.90, −ln⁡(*π*) and *π*/(1 − *π*)  are very close. Hence, for large values of *π*,

(36) $$\frac{\ln\left(\pi_{1}\right)}{\ln\left(\pi_{2}\right)}≅\frac{\pi_{1}/1-\pi_{1}}{\pi_{2}/1-\pi_{2}}=\text{OR}.$$

And from (7), odds ratio is given by

(37) $$\text{OR}=\exp\left(\frac{\pi }{\sqrt{6}}\cdot \frac{\beta_{j}}{\sigma }\right).$$

The parameters estimated from the linear regression can be interpreted as an odds ratio.

## 8. Simulation Study

It should be noted that, as in Table 1, the proposed method is a consistent improvement over the dichotomizing method with respect to relative efficiencies. These results hold true under the assumption that predictor variable has a discrete uniform distribution and that the random variables *E*_*i*_ follow an extreme value distribution. To demonstrate the robustness of this conclusion to changes in the distributions of predictor variables, simulations were run under different distributional conditions. The data were sampled 10000 times for three sample sizes {*n* = 250, 500, 1000}, three average proportions of successes $\left\{\overset{̅}{\pi }=\;0.10,\;0.50,\;0.95\right\}$, and seven *ω*_*j*_{*ω*_*j*_ = 0.75, 0.90, 1.1, 1.2, 1.3, 1.4, 1.5}. The simulated data are generated using the following algorithm

1. Generate *y*_*i*_, where *y*_*i*_ = *β*_0_ + *β*_1_ *x*_*i*_ + *E*_*i*_, $\beta_{1}=\sqrt{6}\ln\omega_{j}/\pi$ through (7) to produce the correct *ω*_*j*_, and for simplicity *β*_0_ = 0, *σ* ^2^ = 1.
2. For fixed $\overset{̅}{\pi }$, generate cutoff point *C* using (15).

We simulated the data for two scenarios based on the distribution of the explanatory variable. In the first scenario, the independent variable follows a continuous uniform distribution and range (−2, 2), and in the second, the independent variable follows a truncated normal distribution with mean 0 and range (−2, 2). The relative mean square errors, relative interval lengths, absolute biases, and the probability of coverage were calculated.

Results of the simulations addressing the validity of the proposed method are displayed in Tables 2 and 3.

The simulations show that the relative mean square errors are all greater than 1, increasing with the average proportion of successes and when the *ω*_*j*_ moves farther away from 1. The results in Tables 1 and 2 demonstrate that the proposed method provides confidence intervals which successfully maintain their nominal 95 percent coverage. For the proposed method in first scenario, 51 out of 63 coverage probabilities fell within (0.94, 0.96), and all 63 coverage probabilities are greater than 0.93 and, in the second scenario, almost all coverage probabilities fell within (0.94, 0.96). The absolute biases for proposed method are never greater than a few percent. The proposed method is less biased than the dichotomizing method in 6 of 63 simulations in both two scenarios.

## 9. An Example

To illustrate the application of the proposed method presented in the previous section, we utilize the data arising from the National Health Survey in Iran. The other analyses using this data appear in many places [16].

In this study, 14176 women aged 20–69 years were investigated. BMI (body mass index), our dependent variable, was calculated as weight in kilograms divided by height in meters squared (kg/m^2^). Independent variables included place of residence, age, smoking, economic index, marital status, and education level. The independent variables considered were both categorical and continuous. At first, BMI was treated as a continuous variable, and ${\hat{\omega }}_{j}$ and 95 percent confidence intervals were calculated using the proposed linear regression method. Then subjects were classified into obese (BMI ≥ 30 kg/m^2^) and nonobese (BMI <30 kg/m^2^). A complementary log-log model was used for the binary analysis, with obese or nonobese used as the outcome measure. The ${\hat{\omega }}_{j}^{*}$ and 95 percent confidence intervals were calculated using the dichotomized method.  Table 4 presents the coefficient estimates, estimated confidence intervals, and relative confidence interval lengths. The proposed and dichotomizing methods produced different confidence intervals, although the ${\hat{\omega }}_{j}$ and ${\hat{\omega }}_{j}^{*}$ were similar only varying slightly. The ${\hat{\omega }}_{j}\;$estimate from the proposed method had smaller variances and shorter confidence intervals than the dichotomizing method. All relative confidence interval lengths were greater than 2.58. 

## 10. Discussion

When assuming the errors  *E*_*i*_  are distributed as an extreme value distribution, as noted before, the method has several advantages. First, the method allows the researcher to apply the complementary log-log model without dichotomizing and without loss of information. Second, the ${\hat{\omega }}_{j}^{*}$ from the dichotomizing method is dependent on the chosen cutoff point *C* and will vary with *c*. However, the proposed ${\hat{\omega }}_{j}$ is independent of the *c* since ${\hat{\omega }}_{j}$ is a function of the continuous *Y*_*i*_  and not a function of the dichotomized *Y*_i_*  defined through *C*. Third, we show that the coefficient of the complementary log-log model, *θ*_*j*_, can be interpreted in terms of the regression coefficients, *β*_*j*_. Fourth, when the independent variables *x*_*i*_ follow a discrete uniform distribution, the proposed method is a consistent improvement over the dichotomizing method with respect to relative efficiencies. The proposed method can provide sample size saving, smaller variances, and shorter confidence intervals than the dichotomized method. Fifth, when *π*  is large, the parameters estimated from the linear regression can be interpreted as odds ratios.

Our results were consistent with the findings by Moser and Coombs [12] and Bakhshi et al. [16] showing the greater efficiency of parameter estimates from the regression method that avoids dichotomizing in comparison with a more traditional dichotomizing method using the logistic regression.

Our main recommendation is to let continuous response remain continuous. Do not throw away information by transforming the data to binary. This means that if the objective is to estimate and/or test coefficients when responses are continuous, please resist dichotomizing your response variable.

## Figures and Tables

**Table 1:** Relative sample sizes required to attain any power for the dichotomizing method versus the proposed method.

ω* = exp⁡(*θ*)	Average proportion of successes ($\overset{̅}{\pi }$)				
	0.1	0.2	0.3	0.4	0.5
0.25	23.7166	9.5092	7.4954	7.1996	6.8575
0.50	10.6719	5.4176	3.4215	2.5209	2.1784
0.75	7.7088	3.8713	2.5171	1.9380	1.5841

**Table 2:** Simulated relative mean square errors, relative intervals lengths, coverage probabilities, and absolute biases for the proposed and dichotomizing methods (using a continuous uniform distribution for the explanatory variable and an extreme value distribution for the errors).

Sample size	*ω* Cut off	.75	.9	1.1	1.2	1.3	1.4	1.5
		1.15^a^	1.07	1.09	1.14	1.24	1.47	1.71
		1.10^b^	1.03	1.03	1.07	1.14	1.23	1.35
	0.10	0.943^c^	0.948	0.949	0.949	0.945	0.938	0.933
		0.948^d^	0.947	0.949	0.947	0.951	0.947	0.953
		0.05^e^	0.04	0.12	0.14	0.10	0.15	0.11
		0.07^f^	0.01	0.17	0.13	0.24	0.34	0.58
								
		1.23	1.26	1.27	1.28	1.27	1.24	1.26
		2.16	1.13	1.23	1.14	1.15	1.17	1.19
1000	0.50	0.940	0.951	0.951	0.945	0.942	0.937	0.934
		0.951	0.949	0.951	0.950	0.948	0.947	0.948
		0.04	0.01	0.08	0.10	0.05	0.09	0.04
		0.05	0.04	0.15	0.12	0.09	0.12	0.13
								
		12.75	12.44	13.22	12.68	13.14	12.91	12.79
		3.67	3.57	3.58	3.63	3.69	3.76	3.84
	0.95	0.943	0.951	0.952	0.944	0.944	0.938	0.929
		0.952	0.954	0.952	0.952	0.951	0.951	0.951
		0.04	0.07	0.11	0.10	0.10	0.17	0.10
		0.75	0.68	0.86	1.01	1.21	1.45	1.24

		1.30	1.08	1.07	1.17	1.24	1.54	1.95
		1.16	1.03	1.04	1.08	1.15	1.25	1.39
	0.10	0.942	0.950	0.951	0.95	0.944	0.941	0.936
		0.951	0.950	0.949	0.951	0.954	0.954	0.953
		0.12	0.07	0.24	0.25	0.21	0.18	0.29
		0.23	0.08	0.33	0.39	0.41	0.73	1.21
								
		1.35	1.10	1.27	1.26	1.26	1.25	1.26
		1.26	1.03	1.13	1.14	1.16	1.17	1.20
500	0.50	0.940	0.949	0.947	0.948	0.943	0.940	0.933
		0.952	0.951	0.949	0.949	0.954	0.950	0.951
		0.23	0.34	0.27	0.23	0.26	0.25	0.38
		0.48	0.11	0.17	0.18	0.31	0.26	0.42
								
		13.04	13.17	13.8	13.90	14.45	14.48	14.47
		3.72	3.65	3.68	3.73	3.82	3.91	3.99
	0.95	0.942	0.947	0.951	0.949	0.947	0.938	0.935
		0.953	0.952	0.954	0.955	0.955	0.953	0.954
		0.05	0.11	0.08	0.08	0.24	0.32	0.27
		0.94	1.38	1.78	1.92	2.52	3.00	2.90

		13.41	14.46	1.12	1.28	1.52	1.96	2.33
		3.78	3.73	1.04	1.09	1.18	1.30	1.45
	0.10	0.942	0.949	0.949	0.945	0.942	0.942	0.933
		0.957	0.954	0.948	0.949	0.952	0.957	0.953
		0.02	0.20	0.38	0.33	0.42	0.41	0.66
		2.11	2.74	0.42	0.84	1.18	1.78	2.24
								
		1.27	1.25	1.32	1.28	1.30	1.30	1.29
		1.16	1.13	1.13	1.14	1.16	1.18	1.20
250	0.50	0.941	0.948	0.952	0.947	0.945	0.943	0.933
		0.951	0.951	0.951	0.950	0.951	0.951	0.951
		0.12	0.13	0.35	0.44	0.41	0.53	0.55
		0.11	0.22	0.39	0.47	0.51	0.74	0.59
								
		12.98	14.6	15.64	15.46	17.05	16.89	18.33
		3.75	3.72	3.82	3.88	4.01	4.12	4.29
	0.95	0.945	0.955	0.946	0.948	0.940	0.937	0.932
		0.959	0.955	0.955	0.959	0.958	0.957	0.952
		0.02	0.16	0.39	0.22	0.46	0.47	0.51
		1.22	2.75	3.97	3.98	4.99	5.19	6.19

a: Relative mean square errors, b: Relative intervals lengths, c: Coverage probability (proposed), d: Coverage probability (dichotomized), e: % bias (proposed), f: % bias (dichotomized).

**Table 3:** Simulated relative mean square errors, relative intervals lengths, coverage probabilities, and absolute biases for the proposed and dichotomizing methods (using a truncated normal distribution for the explanatory variable and an extreme value distribution for the errors).

Sample size	*ω* Cut off	.75	.9	1.1	1.2	1.3	1.4	1.5
		1.17^a^	1.02	1.08	1.13	1.19	1.28	1.36
		1.11^b^	1.03	1.03	1.06	1.10	1.25	1.22
	0.10	0.942^c^	0.948	0.948	0.952	0.944	0.942	0.940
		0.951^d^	0.951	0.950	0.952	0.949	0.951	0.951
		0.08^e^	0.06	0.03	0.14	0.13	0.14	0.16
		0.10^f^	0.11	0.15	0.23	0.30	0.39	0.39
								
		1.26	1.24	1.26	1.28	1.28	1.25	1.28
		1.24	1.13	1.13	1.14	1.14	1.15	1.17
1000	0.50	0.944	0.948	0.952	0.947	0.947	0.944	0.941
		0.948	0.951	0.949	0.949	0.947	0.950	0.949
		0.02	0.09	0.08	0.07	0.18	0.16	0.13
		0.03	0.06	0.12	0.16	0.20	0.16	0.14
								
		12.33	13.12	13.03	12.71	12.86	12.55	12.88
		3.62	3.59	3.61	3.62	3.64	3.68	3.71
	0.95	0.944	0.951	0.948	0.948	0.945	0.945	0.946
		0.952	0.948	0.95	0.949	0.949	0.951	0.952
		0.10	0.04	0.11	0.04	0.16	0.16	0.20
		1.26	1.05	1.56	1.36	1.43	1.80	1.94

		1.18	1.09	1.06	1.75	1.23	1.32	1.58
		1.11	1.03	1.03	1.06	1.11	1.16	1.23
	0.10	0.945	0.95	0.951	0.951	0.949	0.943	0.944
		0.953	0.953	0.953	0.950	0.949	0.951	0.950
		0.04	0.13	0.31	0.18	0.33	0.36	0.37
		0.21	0.08	0.37	0.50	0.62	0.69	0.96
								
		1.25	1.27	1.27	1.29	1.27	1.29	1.25
		1.14	1.13	1.13	1.14	1.15	1.16	1.17
500	0.50	0.944	0.948	0.949	0.947	0.948	0.944	0.935
		0.951	0.951	0.951	0.948	0.951	0.948	0.949
		0.13	0.22	0.35	0.37	0.35	0.30	0.44
		0.16	0.19	0.39	0.48	0.44	0.41	0.54
								
		13.11	14.02	14.02	13.5	13.54	13.80	14.32
		3.73	3.71	3.73	3.75	3.77	3.81	3.86
	0.95	0.944	0.95	0.951	0.950	0.947	0.944	0.944
		0.954	0.95	0.951	0.953	0.948	0.956	0.953
		0.15	0.10	0.24	0.38	0.32	0.33	0.43
		2.50	2.70	2.92	3.10	2.92	3.36	3.89

		1.28	1.11	1.12	1.19	1.33	1.54	1.76
		1.11	1.03	1.04	1.08	1.13	1.19	1.28
	0.10	0.947	0.951	0.950	0.947	0.950	0.950	0.942
		0.951	0.950	0.950	0.952	0.954	0.952	0.951
		0.40	0.34	0.37	0.64	0.69	0.58	0.81
		0.26	0.06	0.69	1.08	1.30	1.55	2.22
								
		1.32	1.30	1.27	1.33	1.31	1.33	1.31
		1.15	1.13	1.13	1.14	1.18	1.17	1.18
250	0.50	0.951	0.95	0.953	0.951	0.940	0.945	0.940
		0.949	0.951	0.952	0.948	0.948	0.950	0.948
		0.22	0.43	0.57	0.69	0.66	0.58	0.66
		0.38	0.53	0.64	0.89	0.91	0.82	0.91
								
		14.09	14.51	16.27	15.91	15.89	15.73	15.60
		3.86	3.87	3.93	3.92	3.98	4.04	4.11
	0.95	0.943	0.95	0.951	0.951	0.947	0.944	0.937
		0.953	0.95	0.953	0.956	0.953	0.956	0.952
		0.30	0.37	0.57	0.68	0.42	0.62	0.75
		4.98	5.52	6.547	5.91	6.17	6.88	7.72

a: Relative mean square errors, b: Relative intervals lengths, c: Coverage probability (proposed), d: Coverage probability (dichotomized), e: % bias (proposed), f: % bias (dichotomized).

**Table 4:** Adjusted ${\hat{\omega }}_{j}^{*}$, ${\hat{\omega }}_{j}\;$for obesity and confidence intervals using two methods for the National Health Survey.

Covariates	${\hat{\omega }}_{j}$(${\hat{\omega }}_{j}^{*}$)	95% CI^a^ (proposed)	95% CI (dichotomized)	Relative^b^ length of CI
Place of residence	1.65 (1.97)^c^	1.58–1.74	1.79–2.18	2.43
Age	1.021 (1.019)	1.018–1.022	1.015–1.022	1.75
Years of education	0.99 (0.98)	0.985–0.997	0.971–0.994	1.92
Smoking	0.76 (0.68)	0.66–0.90	0.51–0.92	1.71
Marital status	1.16 (1.42)	1.10–1.22	1.27–1.58	2.58
Lower-middle economy index	1.24 (1.32)	1.14–1.32	1.18–1.48	1.67
Upper-middle economy index	1.21 (1.26)	1.14–1.29	1.12–1.42	2.0
High economy index	1.20 (1.21)	1.11–1.30	1.08–1.36	1.47

^a^confidence interval, ^b^dichotomized/proposed, ^c^proposed (dichotomized).

## Appendix

### A. Largest Extreme Value Distribution

(a) *The PDF and CDF are given by *

(A.1) $$\begin{array}{cc} f\left(y\;|\;x\beta ,\sigma \right) & =\frac{\pi }{\sigma \sqrt{6}}\\ & \;\;\times \exp(-\frac{y-x\beta -k\sigma }{\sigma }\times \frac{\pi }{\sqrt{6}}\\ & \;\;\;\;\;-\exp\left(\frac{y-x\beta -k\sigma }{\sigma }\times \frac{\pi }{\sqrt{6}}\right))\\ & \;-\infty \langle x\langle \infty ,\sigma \rangle 0,\\ P\left(y\leq c\right) & =1-\exp\left(-\exp\left(-\frac{c-x\beta -k\sigma }{\sigma }\times \frac{\pi }{\sqrt{6}}\right)\right)\\ & \;-\infty \langle x\langle \infty ,\sigma \rangle 0, \end{array}$$

where *Y* is a continuous outcome variable, *x* = (1, *x*_1_,…, *x*_*p*−1_) is the *p* × 1 vector of known independent variables, *β* = (*β*_0_, *β*_1_,…, *β*_*p*−1_) is the *p* × 1 vector of unknown parameters, and *k* ≈ 0.45.

It is easy to check that

(A.2) $$\begin{array}{cc} \omega_{j} & =\frac{\ln\left(1-\pi_{1}\right)}{\ln\left(1-\pi_{2}\right)}=\frac{\ln\left(1-p\left(y\leq c\;|\;x\right)\right)}{\ln\left(1-p\left(y\leq c\;|\;x_{\left(-1,j\right)}\right)\right)}\\ & =\frac{-\exp\left(-\left(\left(c-x^{\prime }\beta -k\sigma \right)/\sigma \right)\times \left(\pi /\sqrt{6}\right)\right)}{-\exp\left(-\left(\left(c-x_{\left(-1,j\right)}^{\prime }\beta -k\sigma \right)/\sigma \right)\times \left(\pi /\sqrt{6}\right)\right)}\\ & =\exp\left(\frac{\pi }{\sqrt{6}}\cdot \frac{\beta_{j}}{\sigma }\right)\Rightarrow 1-\pi_{1}\\ & ={\left(1-\pi_{2}\right)}^{\exp(\left(\pi /\sqrt{6})\cdot (\beta_{j}/\sigma \right))}, \end{array}\;$$

where

(A.3) $$\begin{array}{cc} x_{} & =\left(1,x_{1},\ldots ,x_{j},\ldots ,x_{p-1}\right),\\ x_{\left(-1,j\right)} & =\left(1,x_{1},\ldots ,x_{j}-1,\ldots ,x_{p-1}\right),\\ \beta & ={\left(\beta_{0},\beta_{1},\ldots ,\beta_{j},\ldots ,\beta_{p-1}\right)}^{\prime }. \end{array}$$

(b) *Suppose that E*_*i*_ *is distributed as a largest extreme value with mean 0 and variance σ* ^2^ > 0. We conclude that the PDF and CDF of each independent *Y*_*i*_ are given by (A.1), and the corresponding equality (A.2) is given by

(A.4) $${\hat{\omega }}_{j}=\frac{\ln\left(1-{\hat{\pi }}_{1}\right)}{\ln\left(1-{\hat{\pi }}_{2}\right)}=\exp\left(\frac{\pi }{\sqrt{6}}\cdot \frac{{\hat{\beta }}_{j}}{\hat{\sigma }}\right).$$

(c) *Similar to largest extreme value distribution *

(A.5) $$\begin{array}{cc} \mu_{i} & =\pi_{i}=1-\exp\left(-\exp\left(\sum_{j}\theta_{j}x_{ij}\right)\right)\\ & \Rightarrow \pi_{i}=1-\exp\left(-\exp\left(\eta_{i}\right)\right),\\ \frac{\partial \mu_{i}}{\partial \eta_{i}} & =-{\left(-\exp\left(\eta_{i}\right)\right)}^{\prime }\exp\left(-\exp\left(\eta_{i}\right)\right)\\ & =-\left(1-\pi_{i}\right)\ln\left(1-\pi_{i}\right)\\ w_{i} & =\frac{{\left(\left(1-\pi_{i}\right)\ln\left(1-\pi_{i}\right)\right)}^{2}}{\pi_{i}\left(1-\pi_{i}\right)}\\ & =\frac{\left(1-\pi_{i}\right){\left(\ln\left(1-\pi_{i}\right)\right)}^{2}}{\pi_{i}}, \end{array}$$

then

(A.6) $$X^{\prime }WX=\left[\begin{array}{cccc} \overset{n}{\underset{i=1}{\sum }}\frac{\left(1-\pi_{i}\right){\left(\ln\left(1-\pi_{i}\right)\right)}^{2}}{\pi_{i}} & \overset{n}{\underset{i=1}{\sum }}\frac{\left(1-\pi_{i}\right){\left(\ln\left(1-\pi_{i}\right)\right)}^{2}}{\pi_{i}} & \cdots & \overset{n}{\underset{i=1}{\sum }}x_{i,p-1}\frac{\left(1-\pi_{i}\right){\left(\ln\left(1-\pi_{i}\right)\right)}^{2}}{\pi_{i}}\\ \overset{n}{\underset{i=1}{\sum }}x_{i1}\frac{\left(1-\pi_{i}\right){\left(\ln\left(1-\pi_{i}\right)\right)}^{2}}{\pi_{i}} & \overset{n}{\underset{i=1}{\sum }}x_{i1}^{2}\frac{\left(1-\pi_{i}\right){\left(\ln\left(1-\pi_{i}\right)\right)}^{2}}{\pi_{i}} & \cdots & \overset{n}{\underset{i=1}{\sum }}x_{1}x_{i,p-1}\frac{\left(1-\pi_{i}\right){\left(\ln\left(1-\pi_{i}\right)\right)}^{2}}{\pi_{i}}\\ \vdots & & \vdots & \\ \overset{n}{\underset{i=1}{\sum }}x_{i,p-1}\frac{\left(1-\pi_{i}\right){\left(\ln\left(1-\pi_{i}\right)\right)}^{2}}{\pi_{i}} & \overset{n}{\underset{i=1}{\sum }}x_{i}x_{i,p-1}\frac{\left(1-\pi_{i}\right){\left(\ln\left(1-\pi_{i}\right)\right)}^{2}}{\pi_{i}} & \cdots & \overset{n}{\underset{i=1}{\sum }}x_{i,p-1}^{2}\frac{\left(1-\pi_{i}\right){\left(\ln\left(1-\pi_{i}\right)\right)}^{2}}{\pi_{i}} \end{array}\right].$$
